# Short-term inhibition of TERT induces telomere length-independent cell cycle arrest and apoptotic response in EBV-immortalized and transformed B cells

**DOI:** 10.1038/cddis.2016.425

**Published:** 2016-12-29

**Authors:** Andrea Celeghin, Silvia Giunco, Riccardo Freguja, Manuela Zangrossi, Silvia Nalio, Riccardo Dolcetti, Anita De Rossi

**Affiliations:** 1 Section of Oncology and Immunology, Department of Surgery, Oncology and Gastroenterology, University of Padova, Padova, Italy; 2 Immunology and Molecular Oncology Unit, Istituto Oncologico Veneto (IOV)-IRCCS, Padova, Italy; 3 Cancer Bio-Immunotherapy Unit, CRO-IRCCS, National Cancer Institute, Aviano, Italy; 4 University of Queensland Diamantina Institute, Translational Research Institute, Brisbane, Queensland, Australia

## Abstract

Besides its canonical role in stabilizing telomeres, telomerase reverse transcriptase (TERT) may promote tumorigenesis through extra-telomeric functions. The possible therapeutic effects of BIBR1532 (BIBR), a powerful TERT inhibitor, have been evaluated in different cellular backgrounds, but no data are currently available regarding Epstein–Barr virus (EBV)-driven B-cell malignancies. Our aim was to characterize the biological effects of TERT inhibition by BIBR on EBV-immortalized lymphoblastoid cell lines (LCLs) and fully transformed Burkitt's lymphoma (BL) cell lines. We found that BIBR selectively inhibits telomerase activity in TERT-positive 4134/Late and 4134/TERT+ LCLs and EBV-negative BL41 and EBV-positive BL41/B95.8 BL cell lines. TERT inhibition led to decreased cell proliferation, accumulation of cells in the S-phase and ultimately to increased apoptosis, compared with mock-treated control cells. All these effects occurred within 72 h and were not observed in BIBR-treated TERT-negative 4134/TERT- and U2OS cells. The cell cycle arrest and apoptosis, consequent upon short-term TERT inhibition, were associated with and likely dependent on the activation of the DNA damage response (DDR), highlighted by the increased levels of *γ*H2AX and activation of ATM and ATR pathways. Analyses of the mean and range of telomere lengths and telomere dysfunction-induced foci indicated that DDR after short-term TERT inhibition was not related to telomere dysfunction, thus suggesting that TERT, besides stabilizing telomere, may protect DNA via telomere-independent mechanisms. Notably, TERT-positive LCLs treated with BIBR in combination with fludarabine or cyclophosphamide showed a significant increase in the number of apoptotic cells with respect to those treated with chemotherapeutic agents alone. In conclusion, TERT inhibition impairs cell cycle progression and enhances the pro-apoptotic effects of chemotherapeutic agents in TERT-positive cells. These results support new therapeutic applications of TERT inhibitors in EBV-driven B-cell malignancies.

Telomerase is a ribonucleoprotein complex containing a catalytic protein with telomere-specific reverse transcriptase (TERT) activity, which synthesizes telomeric sequences *de novo* utilizing an internal RNA template. When the telomere reaches a critical length because of end-replication problems of DNA polymerase, cells cease to proliferate and undergo senescence. Maintenance of telomere length by telomerase is critical for overcoming replicative senescence and acquiring unlimited replicative potential.^1, 2^ In humans, TERT is the rate-limiting component of the telomerase complex^3^ and its expression, usually absent in normal somatic cells, is detectable in most cancer cells.^4^

Recent studies have suggested that, besides maintaining telomere length, TERT is involved in other cellular functions of biological relevance.^5^ In fact, *in vitro* evidence indicates that TERT prevents cell cycle arrest and confers protection from apoptosis induced by adverse culture conditions^6^ and DNA-damaging agents,^7^ prevents cell growth arrest induced by retinoic acid in promyelocytic leukemia-derived cell lines,^8^ antagonizes p53-induced apoptosis in Burkitt's lymphoma (BL) cells^9^ and inhibits apoptosis induced by tumor necrosis factor (TNF)-*α*.^10^

TERT expression also affects the latent/lytic status of Epstein–Barr virus (EBV) in EBV-positive B lymphocytes.^11, 12^ EBV is a ubiquitous human gamma herpesvirus causally linked to the development of several malignancies including BL, Hodgkin's lymphoma, post-transplant lymphoprolipherative disorders and AIDS-associated lymphomas.^13^ EBV has a potent transforming capacity, and efficiently *in vitro* induces uncontrolled proliferation of infected B lymphocytes and generate immortalized lymphoblastoid cell lines (LCLs), which are a suitable *in vitro* model of EBV-driven B-cell lymphomas, mainly those arising in immunocompromised patients. Like many other tumors, EBV-associated malignancies maintain their ability to grow indefinitely through inappropriate activation of telomerase. The latent membrane protein 1 (LMP1), the major EBV oncoprotein, activates the TERT promoter at the transcriptional level via nuclear factor kappa B and MAPK/ERK1/2 pathways and increases telomerase activity in B lymphocytes.^14^ In addition, it has been reported that cells newly infected by EBV exhibit signs of telomere dysfunction and chromosomal rearrangements, mainly due to EBV-mediated displacement of shelterin proteins and uncapping problem at telomeres;^15, 16^ however, established LCLs show minimal or no signal of telomere dysfunction and have a stable karyotype.^15, 16^

Given the ample spectrum of critical functions modulated by TERT, its inhibition could represent a promising strategy to improve cancer treatment, regardless of telomere length. In fact, TERT inhibition in different cellular backgrounds is associated with cell growth arrest, induction of apoptosis^7, 17, 18, 19^ and increased sensitivity to ionizing radiation.^18^ Our previous work has demonstrated that TERT inhibition by short hairpin RNA triggers the complete viral lytic cycle and cell death in EBV-positive cells.^12^

In the growing list of promising anticancer drugs, BIBR1532 (BIBR), a synthetic non-nucleoside compound, can be regarded as one of the most potent specific inhibitors of TERT.^20, 21^ This drug targets the catalytic activity of the telomerase enzyme by binding directly to the telomerase core component thereby reducing the affinity for deoxyribonucleotides (dNTPs). The drug's and TERT-binding sites for dNTPs are close or even overlap, thus creating reciprocal steric interference in binding efficiency.^22, 23^ It has been demonstrated that in long-term cultures of human cancer cells of different histological origin, low doses of BIBR can induce a senescence phenotype associated with telomere shortening, which confirms the drug's ability to inhibit canonical TERT activity on telomere.^22, 24, 25, 26, 27^ It has also been demonstrated that short-term treatment with high doses of BIBR induces cytotoxicity in leukemia cells,^27, 28^ most probably by directly inducing telomere dysfunction.^27^ No data are as yet available concerning the effects of BIBR on EBV-immortalized LCLs and transformed BL cell lines.

On these grounds, we carried out this study aimed at characterizing the effects of BIBR in LCLs and BL cell lines. The impact of BIBR combined with fludarabine (FLU) or cyclophosphamide (CY) treatment on LCL viability, cell cycle profile and apoptosis was also evaluated. The study's ultimate aim was to provide a rationale supporting the inclusion of TERT inhibitors in treatment schedules for EBV-driven B-cell malignancies.

## Results

### TERT inhibition by BIBR

**Figure 1:**
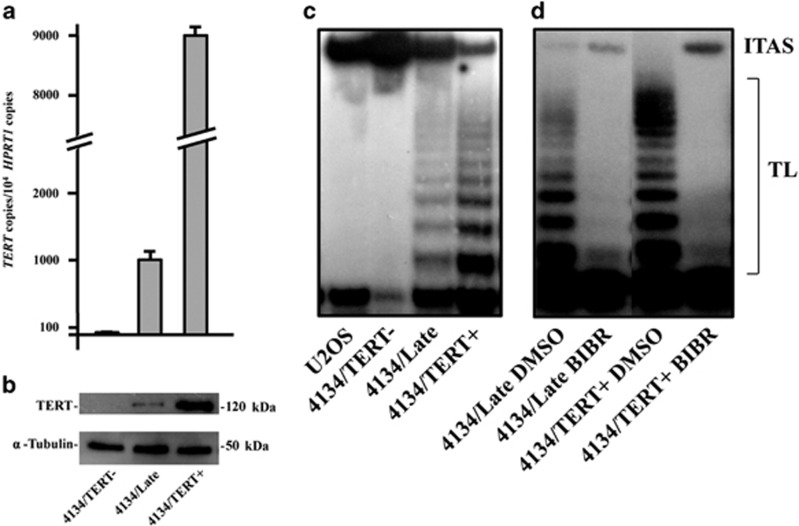
TERT expression and activity in LCLs. (**a**) Levels of TERT transcripts in 4134/TERT-, 4134/Late and 4134/TERT+ LCLs. Means and S.D. (bar) of values from three independent experiments are shown. (**b**) Expression of TERT protein and housekeeping *α*-tubulin in LCLs assessed by western blotting. (**c**) Telomerase activity tested by TRAP assay in telomerase-negative U2OS and 4134/TERT- cells and in telomerase-positive 4134/Late and 4134/TERT+ cells. Panels from one representative experiment are shown. (**d**) *In vitro* efficiency of BIBR tested by TRAP assay in telomerase-positive 4134/Late and 4134/TERT+ by addition of BIBR (2 *μ*M) or DMSO as control in protein extracts. Panels from one representative experiment are shown. TL, telomerase ladder; ITAS, internal telomerase assay standard

4134/Late and 4134/TERT+ LCLs were positive for TERT mRNA, protein expression and telomerase activity, whereas 4134/TERT- cells were not (Figures 1a–c). The telomeric repeat amplification protocol (TRAP) assay, carried out by adding 2 *μ*M BIBR to protein extracts of TERT-positive 4134/Late and 4134/TERT+ cells, demonstrated that BIBR efficiently inhibits telomerase activity in both TERT-positive cell lines (Figure 1d). Similar results were obtained in TERT-positive BL41 and BL41/B95.8 BL cells (data not shown).

The LCLs were then exposed to varying concentrations (from 10 to 60 *μ*M) of BIBR and analyzed for cell viability at 16, 24, 36, 48 and 72 h (Supplementary Figure 1). Treatment with BIBR at 30 *μ*M resulted in decreased proliferation rates of TERT-positive cells at all time points, whereas no effect was seen in TERT-negative 4134/TERT- and U2OS cells. Similar results were reached in the EBV-negative BL41 and its EBV-positive counterpart BL41/B95.8 BL cell lines (Supplementary Figure 1). At 60 *μ*M, even the TERT-negative cell cultures (4134/TERT- and U2OS) showed reduced proliferation rates compared with untreated controls (Supplementary Figure 1). The concentration of 30 *μ*M was then used for experiments in all cell lines.

### BIBR induces S-phase accumulation of TERT-positive LCLs and BL cells

**Figure 2:**
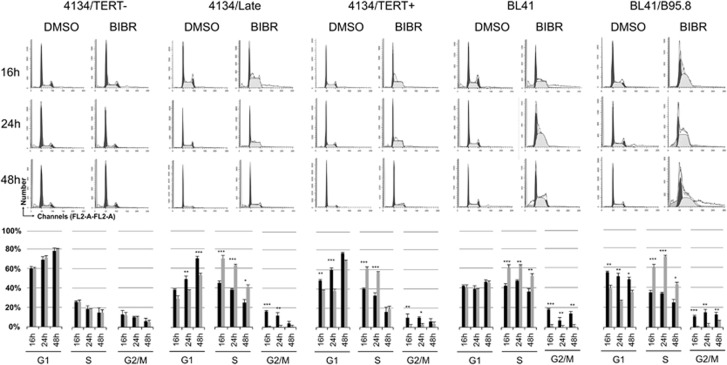
Effect of TERT inhibition by BIBR on cell cycle profiles in LCLs and BL cells. Cells, treated with BIBR (30 *μ*M) and DMSO as control at 16, 24 and 48 h, were labeled with PI and analyzed by flow cytometry for cell cycle distribution. Panels from one representative experiment are shown. Percentages of cells in G1, S and G2/M-phase are shown in graphs below. Black bars: BIBR-treated cells; gray bars: DMSO-treated control cells. Values are means and S.D. (bar) of three separate experiments. Significant differences between values in BIBR-treated *versus* DMSO-treated cells are shown: *P<0.05, **P<0.01 and ***P<0.001

We have previously demonstrated that TERT knockdown by short hairpin RNA induces cell cycle perturbations in both EBV-positive and EBV-negative lymphoma B cells.^12^ TERT-positive 4134/Late and 4134/TERT+ cells treated with BIBR also showed alterations in cell cycle profile, with decreased cells in the G1-phase, disappearance of the G2/M-phase and a significant accumulation of cells in the S-phase (Figure 2). In particular, the S-phase was significantly increased compared with dimethylsulfoxide (DMSO)-treated control cells in both cell cultures, particularly at 16 and 24 h of exposure. Similar findings were observed in both EBV-negative BL41 and EBV-positive BL41/B95.8 BL cells; at 24 h of exposure, both cell lines showed a significant increase of cells in S-phase compared with DMSO-treated control cells (Figure 2). Instead, BIBR treatment did not affect the cell cycle profile of 4134/TERT- (Figure 2) and U2OS cells (Supplementary Figure 2A).

Consistently, the expression of the protein ribonucleotide reductase RNR-R2, a molecular marker of the S-phase, was higher in BIBR-treated TERT-positive LCLs than in untreated controls, whereas 4134/TERT- BIBR-treated cells showed no RNR-R2 upregulation (Supplementary Figure 3). These findings, taken together, support the hypothesis that BIBR can affect cell cycle progression by promoting selective accumulation of cells in the S-phase in TERT-positive B cells.

### TERT inhibition leads to apoptosis in TERT-positive LCLs and BL cells

**Figure 3:**
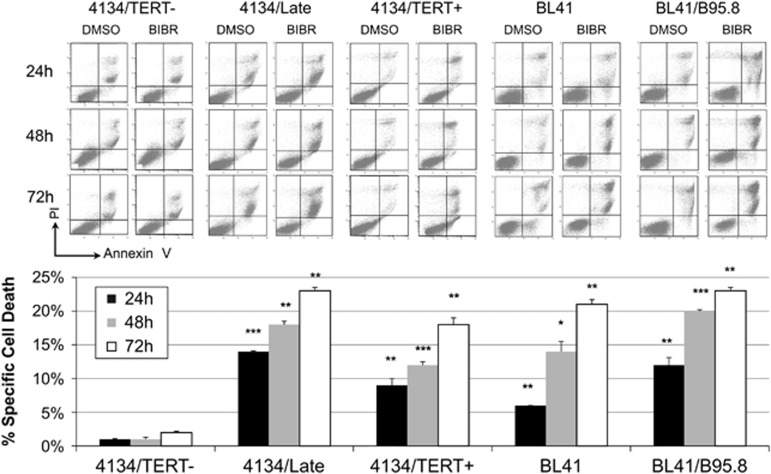
Effect of TERT inhibition by BIBR on cell viability in LCLs and BL cells. Cells treated with BIBR (30 *μ*M) and DMSO at 24, 48 and 72 h, were labeled with annexin V/PI and analyzed by flow cytometry for cell viability. Panels from one representative experiment are shown. Percentages of specific cell death were calculated as described in Materials and Methods section, with DMSO-treated samples as controls. Values are means and S.D. (bar) of three separate experiments. Significant differences between values in BIBR-treated *versus* DMSO-treated cells are shown. *P<0.05, **P<0.01 and ***P<0.001

As previous data had indicated that BIBR can promote apoptosis,^27, 28^ we analyzed the pro-apoptotic effects of this drug in both LCL and BL models. TERT-positive LCLs treated with 30 *μ*M BIBR showed a progressive increase in the number of apoptotic cells compared with controls at all the time points considered (Figure 3). Similar results were observed in BL41 and BL41/B95.8 cells treated with BIBR; a significant increase in the number of apoptotic cells compared with controls was observed at 48 h of exposure, and the rate of apoptotic cells was higher in BL41/B95.8 than BL41 cells (Figure 3). Conversely, 4134/TERT- (Figure 3) and U2OS cells (Supplementary Figure 2B) exposed to BIBR showed no increase in the number of apoptotic cells.

### TERT inhibition activates the ATM/ATR cascade

**Figure 4:**
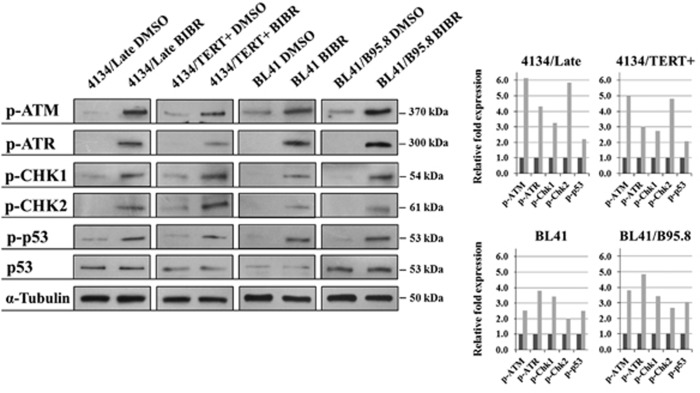
TERT inhibition activates the ATM and ATR cascades. TERT inhibition by BIBR results in activation of ATM/ATR pathways in 4134/Late, 4134/TERT+, BL41 and BL41/B95.8 cell lines. Cells were treated with BIBR (30 *μ*M) and analyzed after 36 h of exposure by western blot. Phospho-ATM (p-ATM), phospho-ATR (p-ATR), phospho-CHK1 (p-CHK1), phospho-CHK2 (p-CHK2), phospho-p53 (p-p53) and p53 (p53) protein expression, detected by specific antibodies, are shown. Graphs on right: densitometry analysis in arbitrary units performed with ImageJ software (NIH, Bethesda, MD, USA), with value of 1 assigned to DMSO-treated control samples. Gray bars: BIBR-treated cells; black bars: DMSO-treated control cells

To shed light on the possible mechanism underlying the cell cycle arrest and apoptosis consequent upon TERT inhibition by BIBR, we studied the involvement of the ATM and ATR pathways, which are critical regulators of cell cycle progression and apoptosis. BIBR treatment resulted in increased levels of the phosphorylated active form of ATM and ATR and their downstream substrates CHK1, CHK2 and pro-apoptotic p53 protein in 4134/Late and 4134/TERT+ cells, as well as in both EBV-negative BL41 and EBV-positive BL41/B95.8 cell lines (Figure 4). Conversely, no changes in the phosphorylation level of these proteins were noted in 4134/TERT- and U2OS cells (Supplementary Figure 4). Thus, TERT inhibition activates ATM and ATR cascades in TERT-positive LCLs and BL cells.

### TERT inhibition leads to H2AX activation in TERT-positive LCLs and BL cells

**Figure 5:**
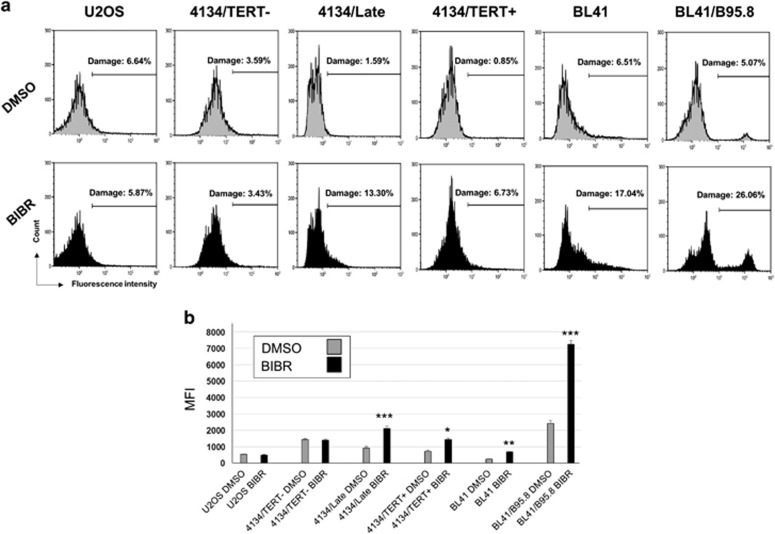
TERT inhibition by BIBR increases DNA damage in TERT-positive cells. (**a**) TERT-positive 4134/Late, 4134/TERT+, BL41 and BL41/B95.8 cells and TERT-negative 4134/TERT- and U2OS cells exposed for 24 h to BIBR or to DMSO as control, were stained with *γ*H2AX to evaluate DNA damage and analyzed by flow cytometry. Panels from one representative experiment are shown. (**b**) Levels of *γ*H2AX MFI in BIBR- and DMSO-treated cells. Significant differences between values in BIBR-treated *versus* DMSO-treated cells are shown: *P<0.05, **P<0.01 and ***P<0.001

ATM and ATR are the key sensors of DNA damage.^29^ Findings that both these proteins are activated in BIBR-treated TERT-positive cells suggested that TERT inhibition could induce DNA damage and activate the DNA damage response (DDR). To assess this possibility, cells were stained for *γ*H2AX, a marker of DNA damage.^30^ As shown in Figure 5a, TERT-positive 4134/Late, 4134/TERT+, BL41 and BL41/B95.8 cells showed a significant increase in *γ*H2AX-positive cells, even after 24 h of exposure (Figure 5a). Conversely, 4134/TERT- and U2OS cells exposed to BIBR showed no evidence of increased DNA damage. The *γ*H2AX mean fluorescence intensity (MFI) also increased significantly in TERT-positive BIBR-treated cell lines compared with DMSO-treated control ones, whereas in TERT-negative cells no differences in MFI levels were observed between cells treated with BIBR or DMSO (Figure 5b).

### Short-term inhibition of TERT does not affect telomere

**Figure 6:**
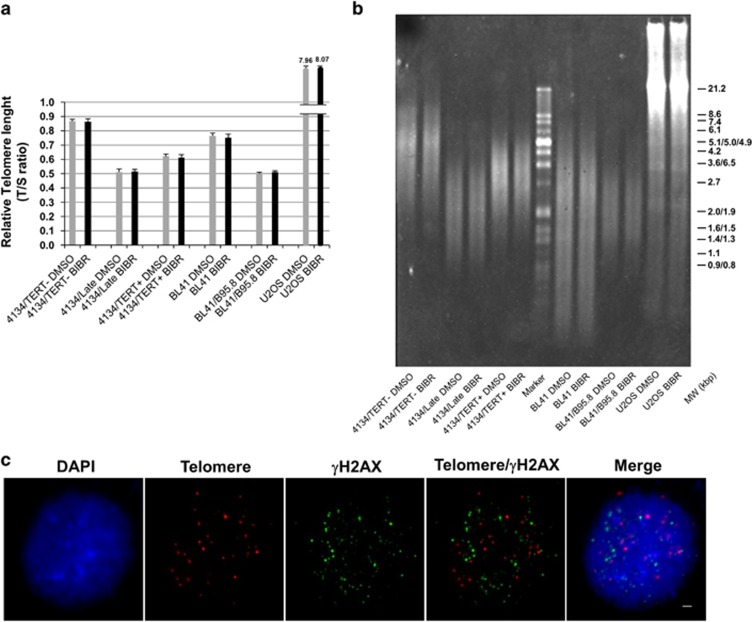
Short-term TERT inhibition by BIBR did not affect telomere. TERT-positive 4134/Late, 4134/TERT+, BL41 and BL41/B95.8 cells and TERT-negative 4134/TERT- and U2OS cells exposed for 72 h to BIBR or to DMSO as control were analyzed for telomere length. (**a**) Telomere length measured by quantitative multiplex PCR assay. TS values are e means and S.D. (bar) of three separate experiments. (**b**) Telomere lengths analyzed by TRF by the TeloTAGGG telomere length assay. Panel from one representative experiment is shown. (**c**) TIF analysis. Representative micrographs showing combined telomere FISH/γH2AX immunofluorescence of 4134/Late cells treated with BIBR at 24 h. From the left: DAPI (nuclear marker, blue), telomere probe (red), γH2AX (DNA damage marker, green), combined Telomere/γH2AX and the merged image. Scale bar: 2 *μ*m

Replicative telomere attrition leads to activation of ATM and ATR. To elucidate whether the DDR in BIBR-treated cells can be activated by telomere erosion, we assessed the effects of the drug on telomere length. BIBR treatment did not affect the telomere length of LCLs or BL cells, as measured by quantitative multiplex PCR at 72 h of exposure (Figure 6a). This finding was confirmed by terminal restriction fragment (TRF) analysis (Figure 6b). Unlike the PCR-based assay, which gives a mean estimate of telomere length of the cellular population, TRF makes it possible to visualize the range of telomere length. The results showed that the TRF ranges are the same in cells treated with BIBR or DMSO, thus excluding the possibility that BIBR selectively targets cells with short telomeres within one cellular population.

It has been demonstrated that EBV infection may cause telomere dysfunction, mainly due to reduction and displacement of TRF2 shelterin protein from telomeres; however, this effect was greatly reduced in LCLs kept in culture for an extended period of time.^15, 16^ In agreement with these observations in established LCLs, in our 4134/Late cells, combined telomere FISH/TRF2 immunofluorescence showed that TRF2 was expressed and localized on telomeres (Supplementary Figure 5B). In addition, the treatment of 4134/Late cells with 30 *μ*M BIBR at 24 h did not modify the expression and localization of TRF2 protein compared with DMSO control cells (Supplementary Figures 5A and 5B). These results suggest that DDR is not driven by TRF2 displacement and uncapping problems at telomeres. To elucidate in greater detail whether DNA damage is associated with telomeres, we examined the presence of telomere dysfunction-induced foci (TIF) in cells exposed to BIBR. Most of the *γ*H2AX foci, markers of DNA damage, did not colocalize with telomere probe signals, and the number of TIF *per* nucleus was always lower than 3 (Figure 6c). All together, these findings indicate that inhibition of TERT by BIBR may lead to DNA damage randomly rather than specifically on telomeres.

### Effects of combined treatment with BIBR and FLU or CY

The observation that TERT inhibition by BIBR leads to cell cycle arrest and apoptosis prompted us to investigate whether TERT inhibition increases susceptibility to antineoplastic drugs. We therefore examined the effects of BIBR in combination with FLU or CY, two of the agents most frequently used to treat B-cell malignancies, in the LCL model.

**Figure 7:**
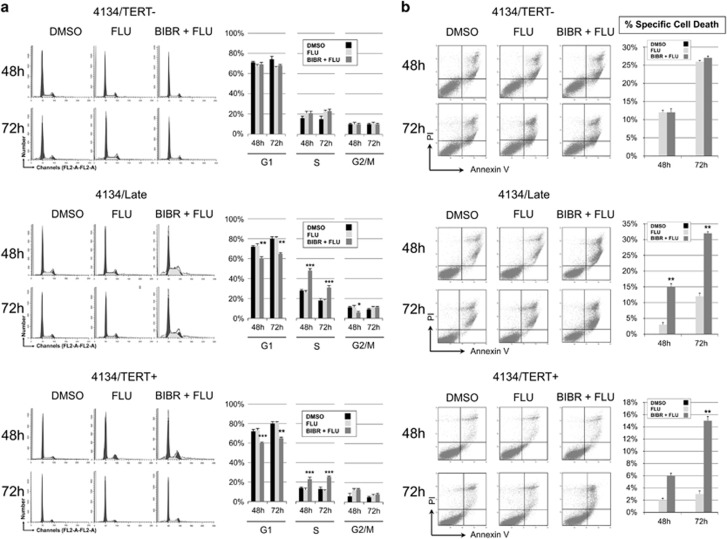
Effects of FLU and BIBR+FLU treatments on cell cycle profiles and cell viability in LCLs. Cells were treated with FLU (5 *μ*M) and BIBR (30 *μ*M) *plus* FLU (5 *μ*M) (BIBR+FLU) and analyzed at 48 and 72 h. DMSO was used as control. (**a**) Cells were labeled with PI and analyzed by flow cytometry. Panels from one representative experiment are shown. Graphs on right: percentages of cells in G1-, S- and G2/M-phase. Values are means and S.D. (bar) of three independent experiments. (**b**) Cells were labeled with annexin V/PI and analyzed by flow cytometry. Panels from one representative experiment are shown. Graphs on right: percentages of specific cell death. Values are means and S.D. (bar) of three separate experiments. Significant differences between values in BIBR+FLU-treated *versus* FLU-treated cells are shown: *P<0.05, **P<0.01 and ***P<0.001

Each drug was used alone or in combination with BIBR. Cells exposed to FLU were analyzed at 48 and 72 h (Figure 7a). In both 4134/Late and 4134/TERT+, treatment with FLU alone did not modify the cell cycle profile, whereas cells treated with BIBR+FLU showed a significant increase of cells in the S-phase and a decrease in the G1-phase, at both 48 and 72 h. Conversely, in 4134/TERT- cells, neither treatments with FLU alone or FLU+BIBR induced significant cell cycle changes (Figure 7a).

Cells treated with FLU and FLU+BIBR were also analyzed for apoptosis. 4134/TERT- cells were more sensitive to FLU alone (apoptosis of 29±2% at 72 h) than 4134/Late cells (14±2% at 72 h) and 4134/TERT+ cells (3±1% at 72 h) (Figure 7b). In 4134/Late and 4134/TERT+ cells, BIBR+FLU treatment significantly increased the percentage of apoptotic cells compared with that obtained with FLU alone. In contrast, BIBR+FLU treatment of TERT-negative cells did not increase the number of apoptotic cells compared with that obtained with FLU alone (Figure 7b).

**Figure 8:**
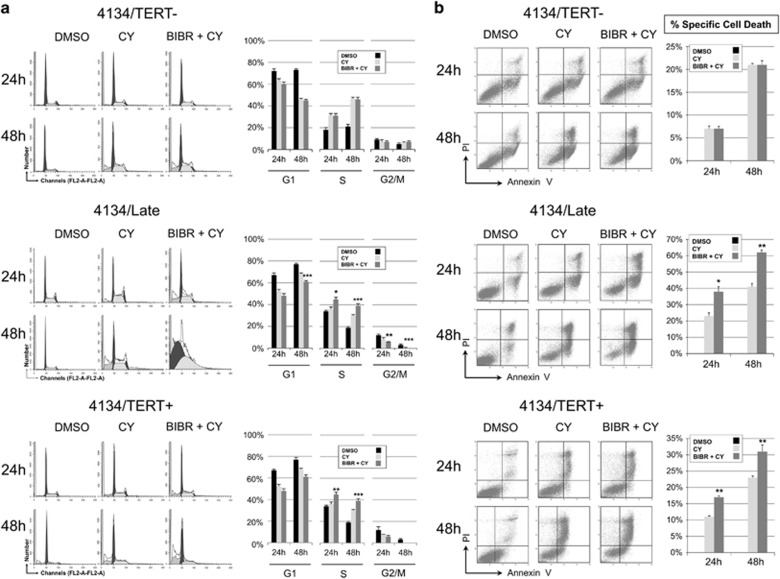
Effects of CY and BIBR+CY treatments on cell cycle profiles and cell viability in LCLs. Cells were treated with CY (4 mM) and BIBR (30 *μ*M) *plus* CY (4 mM) (BIBR+CY) and analyzed at 24 and 48 h. DMSO was used as control. (**a**) Cells were labeled with PI and analyzed by flow cytometry. Panels from one representative experiment are shown. Graphs on right: percentages of cells in G1-, S- and G2/M-phase. Values are means and S.D. (bar) of three separate experiments. (**b**) Cells were labeled with annexin V/PI and analyzed by flow cytometry. Panels from one representative experiment are shown. Graphs on right: percentages of specific cell death. Values are means and S.D. (bar) of three independent experiments. Significant differences between values in BIBR+CY-treated *versus* CY-treated cells are shown: *P<0.05, **P<0.01 and ***P<0.001

Parallel experiments were performed with CY alone or in combination with BIBR. Cell cycle profiles were analyzed at 24 and 48 h in all LCLs (Figure 8a). In 4134/Late and 4134/TERT+, CY induced a decrease of cell number in G1-phase at 48 h, whereas in 4134/TERT- cells CY treatment slightly increased cell number in the S-phase. Treatment with BIBR+CY in TERT-positive cells induced complete arrest of the cell cycle, whereas in 4134/TERT- the pattern observed with BIBR+CY had no effect compared with cell cultures treated with CY alone (Figure 8a). The number of apoptotic cells after exposure to CY alone was higher in 4134/Late (47±4% at 48 h) than in 4134/TERT- cells (21±2%) (Figure 8b). In 4134/Late and 4134/TERT+ cells treatment with BIBR+CY significantly increased the apoptotic effect compared with those obtained with CY alone, whereas BIBR+CY did not change the number of apoptotic cells compared with that obtained with CY alone in 4134/TERT- cell culture (Figure 8b).

## Discussion

In this study, we demonstrate that in TERT-positive LCLs short-term TERT inhibition by BIBR causes cell cycle arrest, accumulation of cells in the S-phase and apoptosis. Similar results were obtained in the BL41 and its EBV convertant BL41/B95.8 BL cell lines. These effects driven by BIBR were telomerase-specific, as they were not observed in telomerase-negative LCL 4134/TERT- and U2OS cells.

This study provides evidence indicating that cell cycle arrest and apoptosis induced by BIBR-mediated TERT inhibition are related and probably dependent on the activation of the DDR pathway. In particular, TERT inhibition induces DNA damage, highlighted by increased levels of γH2AX, resulting in the activation of DDR and phosphorylation of the ATM and ATR kinases, which in turn activate the mitotic checkpoints CHK1, CHK2 and the pro-apoptotic p53 protein to induce cell cycle arrest with accumulation of cells in the S-phase and apoptosis. Notably, in the EBV-positive BL41/B95.8 cells, the inhibition of TERT by BIBR leads to an earlier and greater accumulation of cells in the S-phase, as well as a higher number of apoptotic cells, than in the EBV-negative counterpart BL41 cells. This effect may be due to the underlying EBV infection and in particular to the effects consequent upon TERT inhibition in this cellular background. In fact, it has been demonstrated that the EBV protein BGLF4 can directly promote elongation of the S-phase.^31^ Intriguingly, this protein is expressed during the EBV lytic cycle and we have previously demonstrated that TERT inhibition in EBV-infected cells triggers a complete viral lytic replication.^12^ From a therapeutic perspective, these findings suggest that TERT inhibition may induce more pronounced effects of potential relevance in EBV-associated lymphoproliferations as compared with EBV-unrelated B-cell malignancies.

It is well-known that shelterin proteins binding to telomeres enable cells to distinguish their chromosome ends from DNA breaks and to repress DNA repair reactions.^32, 33^ Replicative telomere attrition with depletion of TRF2 and POT1 shelterin proteins leads to activation of both ATM- and ATR-mediated DDR.^34^ Notably, it has been demonstrated that EBV in newly infected cells may cause telomere dysfunction, mainly due to decreased expression of shelterin proteins and displacement of TRF2 from telomeres;^15, 16^ in addition, the EBV-encoded LMP1 transfected in EBV-negative BL cells promotes downregulation of shelterin proteins.^35^ However, in agreement with previous observations on established LCLs,^15, 16^ we did not find any TRF2 displacement from telomeres in our LCL cells. Nakashima *et al.*^24^ have reported that long-term BIBR treatment of HeLa-EM2-11ht cells is associated with telomere shortening and activation of DDR at telomeres. Telomere shortening after long-term BIBR treatment has also been reported in chronic myeloid leukemia cells^25^ and in human promyelocytic leukemia cells.^26^ Besides these results supporting the ability of BIBR to inhibit the canonical TERT activity on telomeres during long-term treatment, it has been reported that high doses of BIBR induced growth arrest and apoptosis in short-term culture assays in both leukemia cell lines and primary cells from patients with acute myeloid leukemia and chronic lymphocytic leukemia.^27^ Notably, similar findings were also observed in cells without detectable telomerase activity, and the authors suggested that they were due to direct damage by high doses of BIBR on telomere structures, being thus independent of telomerase.^27^

In our *in vitro* models, the DDR pathway activated after short-term exposure to low doses of BIBR seemed to be substantially unrelated to telomere dysfunction, being instead dependent on TERT inhibition *per se*, as none of above effects were observed in TERT-negative cells. BIBR-treated cells have exactly the same mean telomere length, estimated by multiplex PCR, and range, estimated by TRF analysis, as control DMSO-treated cells. In addition, BIBR treatment did not modify the expression and telomere localization of TRF2, which is compatible with the persistence of its capping function on telomeres. The diffuse localization of *γ*H2AX foci and the limited number of TIF in BIBR-treated cells clearly demonstrated that the DNA damage induced by TERT inhibition in short-term experiments was randomly rather than specifically localized on telomeres.

Thus, the findings that TERT inhibition determines DNA damage, unrelated to telomere dysfunction, reinforces the concept that TERT may have additional roles other than maintaining telomere length, and are in line with the growing body of data describing the extra-telomeric functions of telomerase in many biological processes, including cellular proliferation, gene expression regulation, DNA repair process and mitochondrial functionality.^5, 36^ In particular, several lines of evidence demonstrate that TERT is partially targeted to mitochondria, in which it may influence the production of reactive oxygen species (ROS), and thus DNA damage and apoptosis.^37, 38, 39^ TERT is also involved in DNA repair processes,^40, 41^ and TERT inhibition may lead to perturbation of chromatin structure with diminished capacity for DNA repair and thus accumulation of DNA damage.^41^ On these grounds, the DNA damage we observed after short-term TERT inhibition may be due to increased ROS levels and/or perturbation of the chromatin structure. Further studies are warranted to define the mechanisms underlying the short-term consequences of TERT inhibition unrelated to telomere dysfunction.

In the light of the possible integration of TERT inhibitors in chemotherapeutic regimens, we treated the LCLs with two drugs used in manage lymphoproliferative disorders (FLU and CY), both alone and in combination with BIBR. Notably, treatment with FLU alone did not alter the cell cycle profile and induced more pronounced apoptotic effects in TERT-negative than in TERT-positive cells. These observations support the finding that high TERT levels confer protection against apoptosis.^7, 26^ Indeed, TERT inhibition does sensitize cells to the drug-induced apoptotic effect, as demonstrated by the high number of apoptotic cells induced by BIBR+FLU in TERT-positive cell cultures. Consistently, the percentage of apoptotic cells in 4134/Late culture treated with BIBR+FLU at 72 h was similar to that observed in 4134/TERT- cell culture treated with FLU alone.

CY alone induced stronger apoptotic effects in TERT-positive than in TERT-negative cells; this is consistent with its effect in proliferating cells, taking into account the fact that TERT-positive cells proliferated more rapidly than TERT-negative cells. Nonetheless, the addition of BIBR caused cell cycle arrest and an increased apoptotic effect in TERT-positive cells.

Our findings support the concept that inhibition of the extra-telomeric functions of TERT could be exploited as an effective therapeutic strategy for a variety of tumors, including B-cell malignancies, regardless of telomere length. The inclusion of telomerase inhibitors in chemotherapy protocols for cancer patients may have strong effects on cell proliferation and survival and thus may represent a valid strategy to complement current treatment modalities, as also suggested by others.^42, 43^ Confirmation of these findings in primary tumors cells from patients with EBV-driven and unrelated B-cell malignancies and in suitable animal models will pave the way for a solidly based pre-clinical rationale for including TERT inhibitors in chemotherapy protocols for the treatment of these malignancies.

## Materials and methods

### Cell lines

The 4134 LCL was obtained by infecting peripheral blood mononuclear cells from normal donor with the B95.8 EBV strain. Establishment and characterization of this cell line has already been described.^11^ 4134/TERT- and 4134/Late cells were derived from early and late passages after EBV infection and expressed very low and high level of endogenous TERT, respectively.^11, 44^ The 4134/TERT+ cell line, expressing ectopic TERT, was obtained by infecting 4134/TERT- cells with a retroviral vector.^11^ All three 4134 cell lines used in this study were negative for BZLF1 and viral lytic proteins EA-D and gp350. BL41 is an EBV-negative BL cell line with translocated *MYC* gene (kindly provided by Martin Rowe, Cancer Center, University of Birmingham, Birmingham, UK). BL41/B95.8 is the counterpart cell line infected *in vitro* with the B95.8 EBV strain (kindly provided by Martin Allday, Ludwig Institute for Cancer Research, London, UK). LCLs and BL41 were cultured in RPMI-1640 medium (Euroclone, Milano, Italy), supplemented with glutamine 4 mM, 50 mg/ml gentamycin (Sigma-Aldrich, St. Louis, MO, USA) and 10% heat-inactivated fetal bovine serum FBS (Gibco, Milano, Italy; standard medium) at 37 °C and 5% CO_2_. BL41/B95.8 cells were grown in standard medium supplemented with 1 mM sodium pyruvate, 1% nonessential amino acids (Sigma-Aldrich), and 50 mM *β*-mercaptoethanol. The human osteosarcoma cell line U2OS was used as TERT-negative control;^45, 46^ cells were maintained in McCoy's 5A medium modified (Thermo Fisher Scientific, Waltham, MA, USA), supplemented with 10% fetal bovine serum (Gibco). Cell lines were checked and controlled by cytogenetic analyses. All cell lines were tested and resulted negative for mycoplasma contamination.

### Compounds

A stock solution of BIBR (Selleck Chemicals LLC, Houston, TX, USA) at a concentration of 10 mM was prepared by dissolving the compound in sterile DMSO, divided into aliquots and stored at −80 °C until use. FLU (F9813; Sigma-Aldrich) was prepared by resuspending the compound in DMSO at a concentration of 10 mM, divided into aliquots and stored at −20 °C until use. CY (C0768; Sigma-Aldrich) was prepared by dissolving the compound in sodium chloride 0.9% solution at a concentration of 360 mM, divided into aliquots and stored at 4 °C. It was warmed to 37 °C for 30 s, immediately before use.

LCLs were exposed to serial dilution of FLU and CY to identify the half-maximal inhibitory concentration (IC50) (Supplementary Figure 6). Optimal molarity was defined on the basis of the observed effects on the most sensitive cell line to each drug. FLU for 4134/TERT- had an IC50 concentration of 5 *μ*M. CY on 4134/Late exhibited 50% of cell survival at a concentration of 4 mM. These concentrations were used for all drug experiments.

### Real-time PCR for quantification of TERT transcripts

Cellular RNA was extracted and retrotranscribed into cDNA, as previously detailed.^11^ TERT transcripts were quantified by real-time PCR, with the AT1/AT2 primer pair, as previously described.^11, 47^

### Analysis of telomerase activity

For each sample, three million cells were lysed in 50 *μ*l of CHAPS buffer (0.5% CHAPS, 10 mM TrisHCl, pH 7.5, 1 mM MgCl2, 1 mM EGTA, 0.1 mM phenylmethyl-sulfonyl fluoride, 5 mM *β*-mercaptoethanol, 10% glycerol) and incubated at 4 °C for 30 min. The lysate was then centrifuged at 12000 *g* for 30 min at 4 °C and the supernatant collected. Telomerase activity was assessed by the PCR-based TRAP, as previously reported.^48^ The TRAP assay was performed with 0.250 *μ*g of total cell lysate.

### Western blotting

Western blot analyses from cell cultures were prepared as previously reported.^49^ The expression of TERT, RNR-R2, TRF2 and *α*-tubulin was evaluated by anti-TERT (ab94523, Abcam, Cambridge, UK), anti-RRM2/RNR-R2 (B-Bridge International, Cupertino, CA, USA), anti-TRF2 (Novus Biological, Littleton, CO, USA) and anti-*α*-tubulin (Sigma-Aldrich) antibodies (Ab), respectively. The ATM and ATR pathways were examined with specific Ab against the phosphorylated/active form of ATM (ab81292, Abcam), ATR (ab178407, Abcam), CHK1 (ab195753, Abcam), CHK2 (ab195929, Abcam), p53 (9284, Cell Signaling, Danvers, MA, USA) and un-phosphorylated form of p53 (sc-6243, Santa Cruz Biotechnology, Dallas, TX, USA). Blots were incubated with an appropriate peroxidase-conjugated secondary antibody (Sigma-Aldrich) and stained with a chemiluminescence detection kit (SuperSignal West Pico Chemiluminescent Substrate, Pierce, Rockford, IL, USA). *α*-Tubulin was used as control for loading.

### Viability, apoptosis and cell cycle analysis

Cell viability was determined by Trypan blue exclusion in a Countess automated cell counter (Invitrogen, Carlsbad, CA, USA). To evaluate cell cycle distribution, cells were harvested and processed as previously described.^12^ Samples were analyzed by flow cytometry (FACS Calibur; Becton-Dickinson, Franklin Lakes, NJ, USA) and cell cycle profiles were analyzed with ModFit LT Cell Cycle Analysis software (version 2.0) (Verity Software House, Topsham, ME, USA). Apoptosis was evaluated by staining cells with annexin V and propidium iodide (PI; Sigma-Aldrich), as previously detailed,^12^ and analyzed by flow cytometry. At least 50 000 events were acquired; data were processed with CellQuestPro software (Becton-Dickinson), and analyzed by Kaluza Analyzing Software v1.2 (Beckman Coulter, Pasadena, CA, USA). Annexin V-positive/PI-negative and annexin V-positive/PI-positive samples were classified as early and late apoptotic cells, respectively; both fractions were considered apoptotic cells. The percentage of specific cell death was estimated with the following formula: % cell death=100 x (percentage of dead cells in treated sample – percentage of dead cells in control)/(100% – percentage dead cells in control).

### Analysis of DDR

Approximately 1 × 10^6^ cells were stained for 1 h in the dark with the labeled monoclonal antibody for *γ*H2AX (Alexa Fluor 488 mouse anti-H2AX (pS139), clone N1-431, Becton-Dickinson). Samples were analyzed by flow cytometry (FACS Calibur, Becton-Dickinson). A total of 30 000 events were collected according to morphological parameters (forward- and side-scatter). Analysis was performed with Kalusa software (Beckman Coulter). The MFI was measured by BD FACSDiva software (Becton-Dickinson).

### Combined FISH/immunofluorescence

4134/Late cells were harvested following standard cytogenetic's procedure. Hypotonic treatment was carried out with 0.075 M KCl at 37 °C for 30 min and the resulting pellets were fixed with Carnoy's fixative (methanol/acetic acid 3 : 1). Slides were prepared by dropping the fixative on to wet glass slides and were left to dry overnight at room temperature. The slides were treated with pepsin 0.5 mg/ml (Sigma-Aldrich) at 37 °C for 15 min. Telomeres were visualized with the Telomere PNA FISH Kit/Cy3 (DAKO, Glostrup, Denmark). After digestion, slides were dehydrated by consecutive 2 min in 80, 96 and 100% ethanol and air-dried. Ten microliters of probe was added and a denaturation step was performed at 80 °C for 5 min, followed by 2 h of hybridization at room temperature in the dark. Post-hybridization washes were done at 65 °C for 5 min and briefly at room temperature in PBS. Slides were then blocked with 0.2% fish gelatin and 0.5% BSA in PBS (PBG buffer).^50^ To visualize TRF2 location slides were incubated for 1 h with a rabbit polyclonal anti-TRF2 antibody (1 : 1000, Novus Biological) in PBG buffer followed by Alexa Fluor 488 anti-rabbit (Thermo Fisher Scientific) in PBG buffer. To visualize DNA damage foci, slides were incubated for 1 h with a mouse monoclonal anti-*γ*H2AX antibody (1 : 1000, Merck Millipore, Darmstadt, Germany) in PBG buffer, followed by Alexa Fluor 488 anti-mouse secondary antibody (Thermo Fisher Scientific) in PBG buffer. After washing, slides were air-dried and mounted with DAPI/antifade solution (250 ng DAPI/aml Antifade Solution, MetaSystems, Altlussheim, Germany). Microscope analysis were carried out on a fluorescence microscope (Zeiss Imager. Z2, Oberkochen, Germany) equipped with a single band filter for DAPI, Cy3 and FITC. Digital images were captured with CCD camera (iAi CV-M4+CL, Rohs, Yokohama, Japan) using ISIS software (MetaSystems, Heidelberg, Germany) and Z-stacking function with EC PLAN-NEUFLUAR × 100 magnification objective. At least 50 nuclei for each condition were scored in three independent experiments.

### Telomere length measurement

Telomere lengths were determined by quantitative multiplex PCR assay as previously described,^51^ and by the TeloTAGGG Telomere Length Assay Kit (Roche Diagnostic GmbH, Basel, Switzerland) according to the manufacturer's instructions.

### Statistical analyses

Statistical analyses were performed with SPSS software version 21 (IBM, Armonk, NY, USA). Results were analyzed with *t*-test, ANOVA and Mann–Whitney test and *P*-values <0.05 were considered significant.
