# Supplementary material for: Short-term inhibition of TERT induces telomere length-independent cell cycle arrest and apoptotic response in EBV-immortalized and transformed B cells
Source: Cell Death Dis. 2016 Dec 29;7(12):e2562–. doi: 10.1038/cddis.2016.425 (PMC5260987; doi:10.1038/cddis.2016.425)

# Short-term inhibition of TERT induces telomere length-independent cell cycle arrest and apoptotic response in EBV-immortalized and transformed B cells

Andrea Celeghin^a^, Silvia Giunco^a^, Riccardo Freguja^a^, Manuela Zangrossi^a^, **Silvia Nalio^b^** Riccardo Dolcetti^c,d^, Anita De Rossi^a,b^

^a^ Section of Oncology and Immunology, Department of Surgery, Oncology and Gastroenterology, University of Padova, Italy; ^b^Istituto Oncologico Veneto (IOV)-IRCCS, Padova, Italy; ^c^Cancer Bio-Immunotherapy Unit, CRO-IRCCS, National Cancer Institute, Aviano, Italy; ^d^University of Queensland Diamantina Institute, Translational Research Institute, Brisbane, Queensland 4102, Australia;

**Supplementary Information**

**Figure 1 Supplementary**

LCLs, BL and U2OS cells, exposed to serial dilution of BIBR or DMSO as control, were analysed at 16, 24, 36, 48 and 72 h for cell viability by trypan blue exclusion. BIBR 30 μM led to decreased proliferation at 72 h of 57% ± 4% in 4134/Late cells, 30% ± 2% in 4134/TERT+ cells, 42% ± 3% in BL41 cells and 57% ± 2% in BL41/B95.8 cells. No effects were observed in TERT-negative 4134/TERT- or U2OS at this concentration.

**Figure 2 Supplementary**

U2OS cells were treated with BIBR (10 and 30 μM) and DMSO as control and analysed at 24 and 48 h. **A** U2OS cells were labeled with PI and cell cycles were analysed by flow cytometry. Panels from one representative experiment are shown. Graphs on the right: percentages of cells in G1-, S-, and G2/M-phase. Values are means and SD (bar) of 3 separate experiments. **B** Cells were labeled with annexin V/PI and analysed by flow cytometry. Panels from one representative experiment are shown. Graphs on the right: percentages of specific cell death. Values are means and SD (bar) of 3 separate experiments.

**Figure 3 Supplementary**

LCLs were treated with BIBR (30 μM) and DMSO as control for 24 h. Expression of RNR-R2 and housekeeping α-Tubulin were assessed by western blot.

**Figure 4 Supplementary**

BIBR treatment did not induce the activation of ATM/ATR pathway in TERT-negative cells. Cells were treated with BIBR (30 µM) and analysed after 36 h. 4134/TERT- and U2OS cells expressed same level of phosphorylated/active form of ATM, ATR and p53 with and without BIBR treatment. Western blotting shows phosphorylated/active forms of ATM, ATR and phosphorylated/unphosphorylated forms of p53 with specific antibodies. Graphs on right: densitometry analysis in arbitrary units performed with ImageJ software (NIH, Bethesda, MD, USA), with value of 1 assigned to DMSO-treated samples. Grey bars: BIBR-treated cells; black bars: DMSO-treated control cells.

**Figure 5 Supplementary**

**A Western blotting shows TRF2 expression in 4134/Late treated with BIBR and DMSO as controls. α-Tubulin was used as control for loading. B Representative micrographs showing combined telomere FISH/TRF2 immunofluorescence of 4134/Late cells treated with BIBR or DMSO at 24h. From the left: DAPI (nuclear marker, blu), telomere probe (red), TRF2 (green), combined Telomere/TRF2 and the merged image. Scale bar: 2 µm.**

**Figure 6 Supplementary**

Effects of serial concentrations of **A** FLU and **B** CY on percentage of cell viability in 4134/TERT-, 4134/Late and 4134/TERT+. IC50 values of FLU and CY were estimated as 5 μM at 72 h and 4 mM at 48 h, respectively.


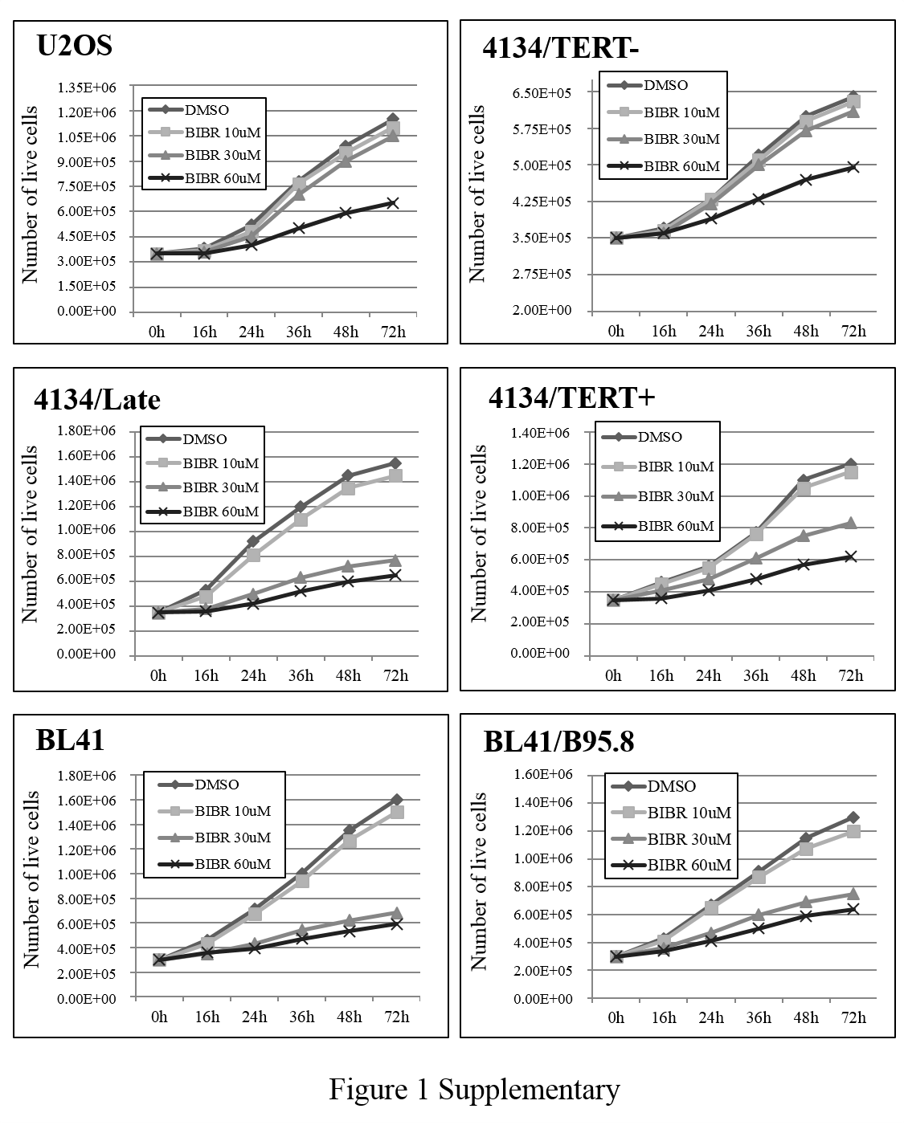

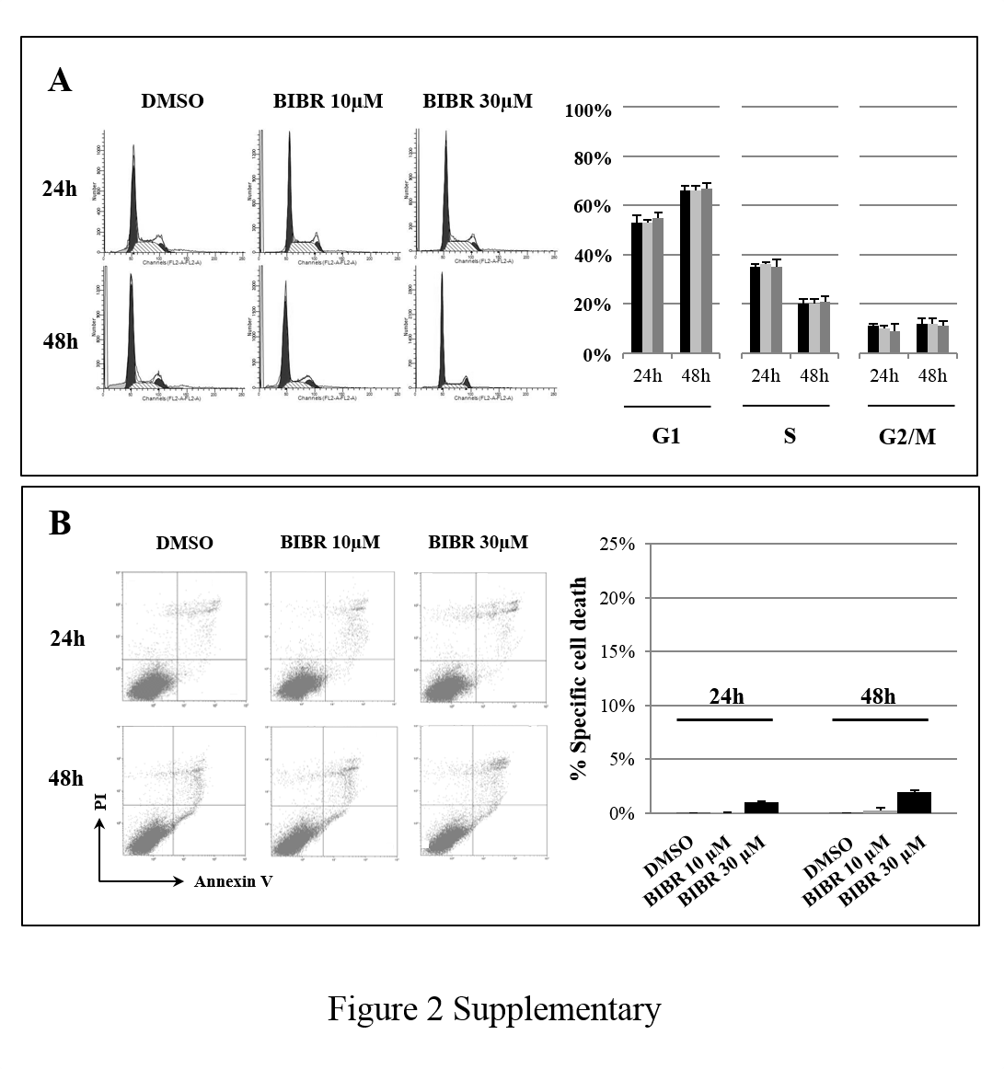

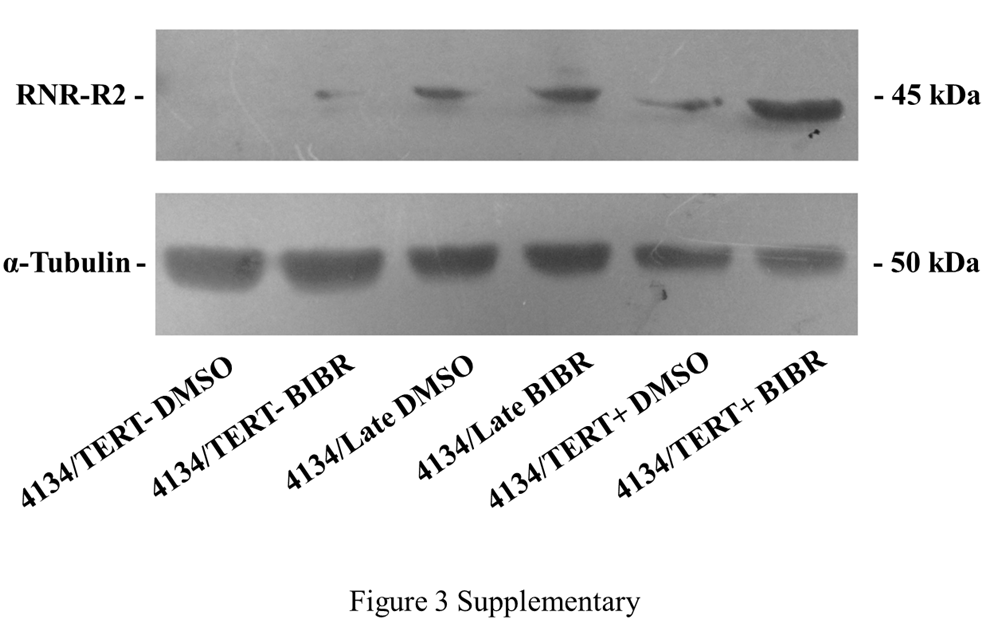

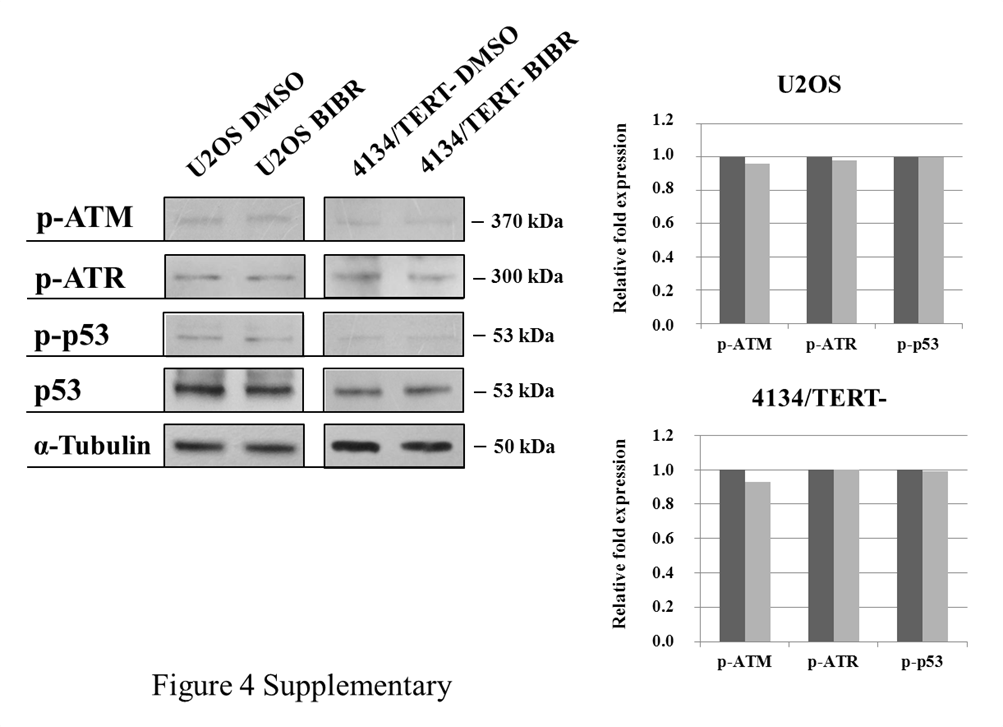

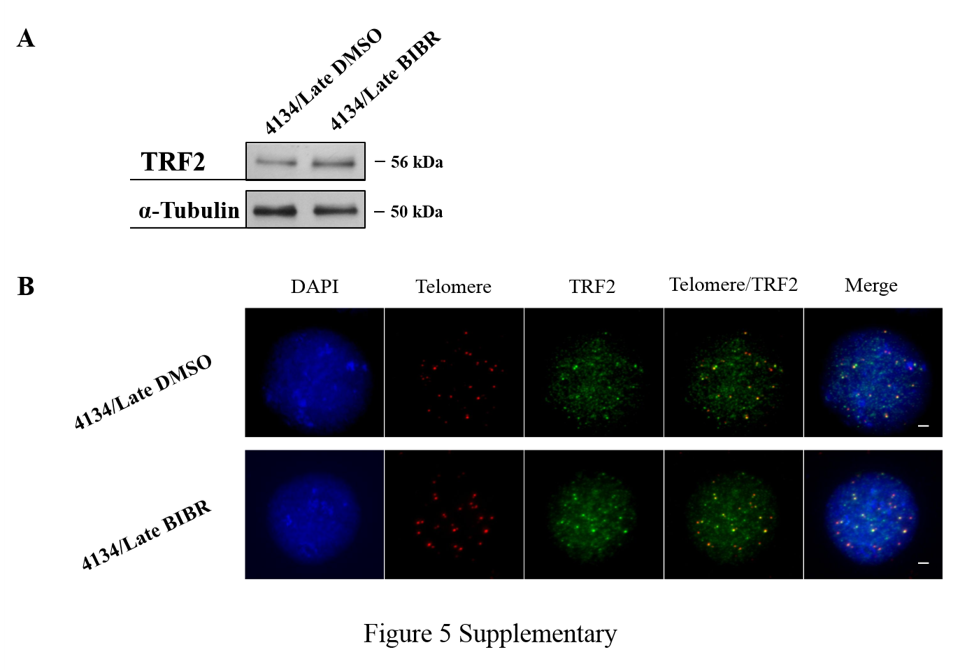

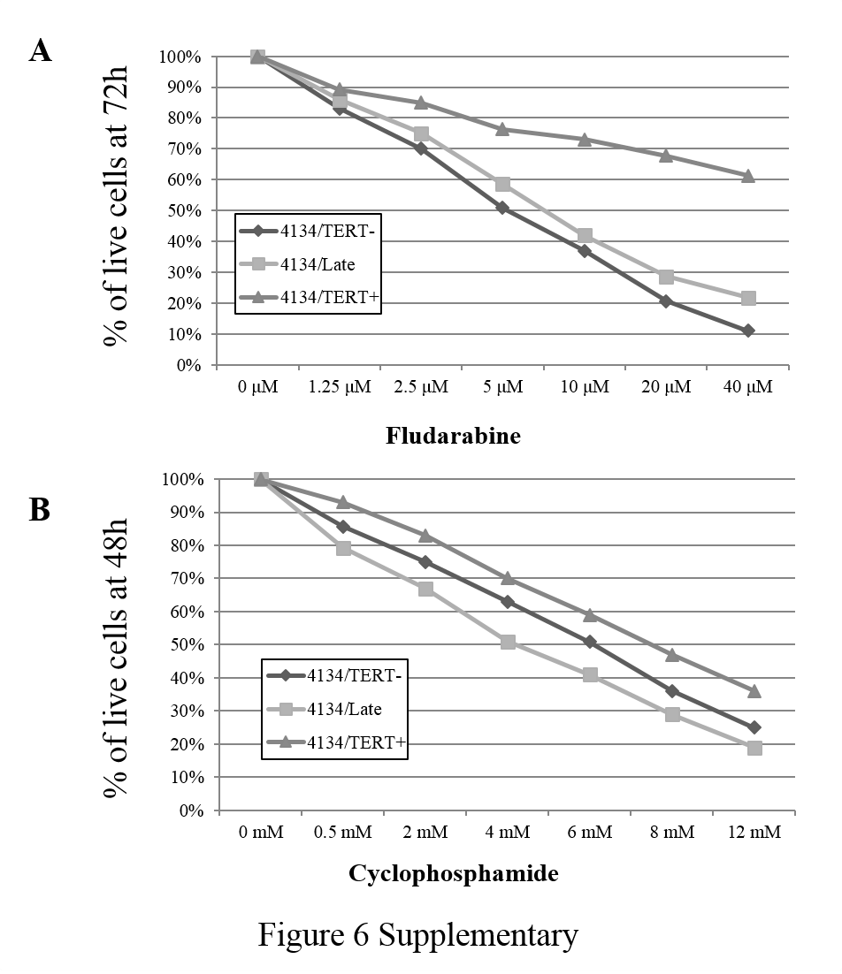

Supplement: Supplementary Information [file cddis2016425x1.docx]
